# Fine mapping of the QTL *cqSPDA2* for chlorophyll content in *Brassica napus* L.

**DOI:** 10.1186/s12870-020-02710-y

**Published:** 2020-11-09

**Authors:** Jingxiu Ye, Haidong Liu, Zhi Zhao, Liang Xu, Kaixiang Li, Dezhi Du

**Affiliations:** State Key Laboratory of Plateau Ecology and Agriculture of Qinghai University, Key Laboratory of Spring Rapeseed Genetic Improvement, Spring Rapeseed Research and Development Center of Qinghai Province, Qinghai Academy of Agricultural and Forestry Sciences, Qinghai University, Xining, 810016 Qinghai China

**Keywords:** *Brassica napus*, Chlorophyll content, Near-isogenic line, Fine mapping, qRT-PCR

## Abstract

**Background:**

Chlorophyll is the most important factor enabling plants to absorb, transfer and transform light energy and plays an important role in yield formation. *Brassica napus* is one of the most important oil crops. Breeding *Brassica napus* for high light efficiency by improving photosynthetic efficiency has considerable social and economic value. In *Brassica napus*, there have been studies of the initial location of chlorophyll in seed embryos and pericarps, but there are few reports on the fine mapping of chlorophyll QTLs. We constructed near-isogenic lines (NIL), fine-mapped a chlorophyll locus, and evaluated the effect of this dominant locus on agronomic traits.

**Results:**

The *cqSPDA2* locus was mapped to an interval of 21.87–22.91 Mb on the chromosome A02 of *Brassica napus* using doubled haploid (DH) lines. To fine-map *cqSPDA2*, we built NIL and designed Indel primers covering the mapping interval. The 469 individuals in the BC_3_F_2_ population were analyzed using these indel primers. Among these indel primers, 15 could narrow the mapping interval to 188 kb between Indel3 and Indel15. Next, 16 indel primers and 19 SSR primers were designed within the new narrower mapping interval, and 5 of the primer-amplified fragments were found to be polymorphic and tightly linked to the *cqSPDA2* locus in the BC_4_F_2_ population. The mapping interval was narrowed to 152 kb on A02 between SSR2 and Indel15. By gene expression analysis, we found three annotated genes in the mapping interval, including *BnaA02g30260D*, *BnaA02g30290D* and *BnaA02g30310D*, which may be responsible for chlorophyll synthesis.

**Conclusions:**

The locus *cqSPDA2*, a dominant QTL for chlorophyll content in *Brassica napus*, was fine-mapped to a 21.89–22.04 Mb interval on A02_._ Three annotated genes (*BnaA02g30260D*, *BnaA02g30290D* and *BnaA02g30310D*) that may be responsible for chlorophyll synthesis were found.

**Supplementary Information:**

The online version contains supplementary material available at 10.1186/s12870-020-02710-y.

## Background

The material basis of crop yield formation is derived from photosynthesis, and high yield based on photosynthesis has long been a hot topic in crop breeding [1]. Chlorophyll is the most important factor enabling plants to absorb, transfer and transform light energy and plays an important role in the growth and development of plants [2]. Maintaining a high level of chlorophyll content in leaves is an important factor in increasing photosynthetic activity [3]. Within a certain range, there is a positive correlation between chlorophyll content and photosynthetic rate, which directly determines the yield [4, 5]. Therefore, chlorophyll content plays an important role in yield formation [6, 7]. Robust seedling development in *Brassica napus* also leads to higher yield stability and has a high importance for plant breeders. Chlorophyll content is a quantitative characteristic that is primarily controlled by nuclear genes and has high heritability [8, 9]. Previous studies have suggested that at least 27 genes are involved in 15 steps of chlorophyll synthesis [10], and biosynthetic defects are considered to be one of the main reasons for low chlorophyll content. Other reasons for chlorophyll deficiency include deficient signal transduction, restrained heme feedback, harmful photooxidation and so on. Overall, the molecular mechanisms of chlorophyll synthesis are very complex [11]. In recent years, researchers have analyzed QTLs for chlorophyll content in the seedling leaves of different populations of multiple crops from different perspectives, making considerable progress and establishing a foundation for future research attempting to elucidate the molecular genetic mechanisms that determine of chlorophyll content [12–18].

The completion of the whole genome sequencing of *Brassica napus* indicates that research on the *Brassica napus* genome has entered a new era. In recent years, with the rapid development of molecular marker technology, it has became possible to construct a high-density molecular marker genetic map of *Brassica napus*. Therefore, the study of chlorophyll QTLs that will allow efficient breeding of *Brassica napus* for greater light efficiency will provide important information for improving the yield potential and direction of high-yield *Brassica napus* breeding in the future. Breeding *Brassica napus* for high light efficiency by improving photosynthetic efficiency is socially and economically important. At present, studies of the initial location of chlorophyll content in the seed embryos [19] and pericarps [20] of winter *Brassica napus* have been conducted, QTLs have been identified under drought and salt stress, and candidate genes related to salt tolerance have even been predicted [21–23]. However, there are few reports of the fine mapping of chlorophyll content QTLs in *Brassica napus* [11].

In a previous study, we discovered a dominant major QTL named *cqSPDA2* located in 21.87–22.91 Mb on A02 using DH lines derived from a cross between Zhongshuang11 (ZS11, a semi-winter variety) and QU (a spring variety), which explained 15.72% variance of phenotype and was detected in six environments stably (under review). In this study, a near- isogenic line (NIL) population for *cqSPDA2* was constructed to further narrow the interval of *cqSPDA2*. This study established a foundation for the cloning of chlorophyll genes controlling photosynthetic function and provided a theoretical basis for improving germplasm resources and selecting new high-yield varieties with molecular markers.

## Results

### Phenotypic and genetic analysis

**Fig. 1:**
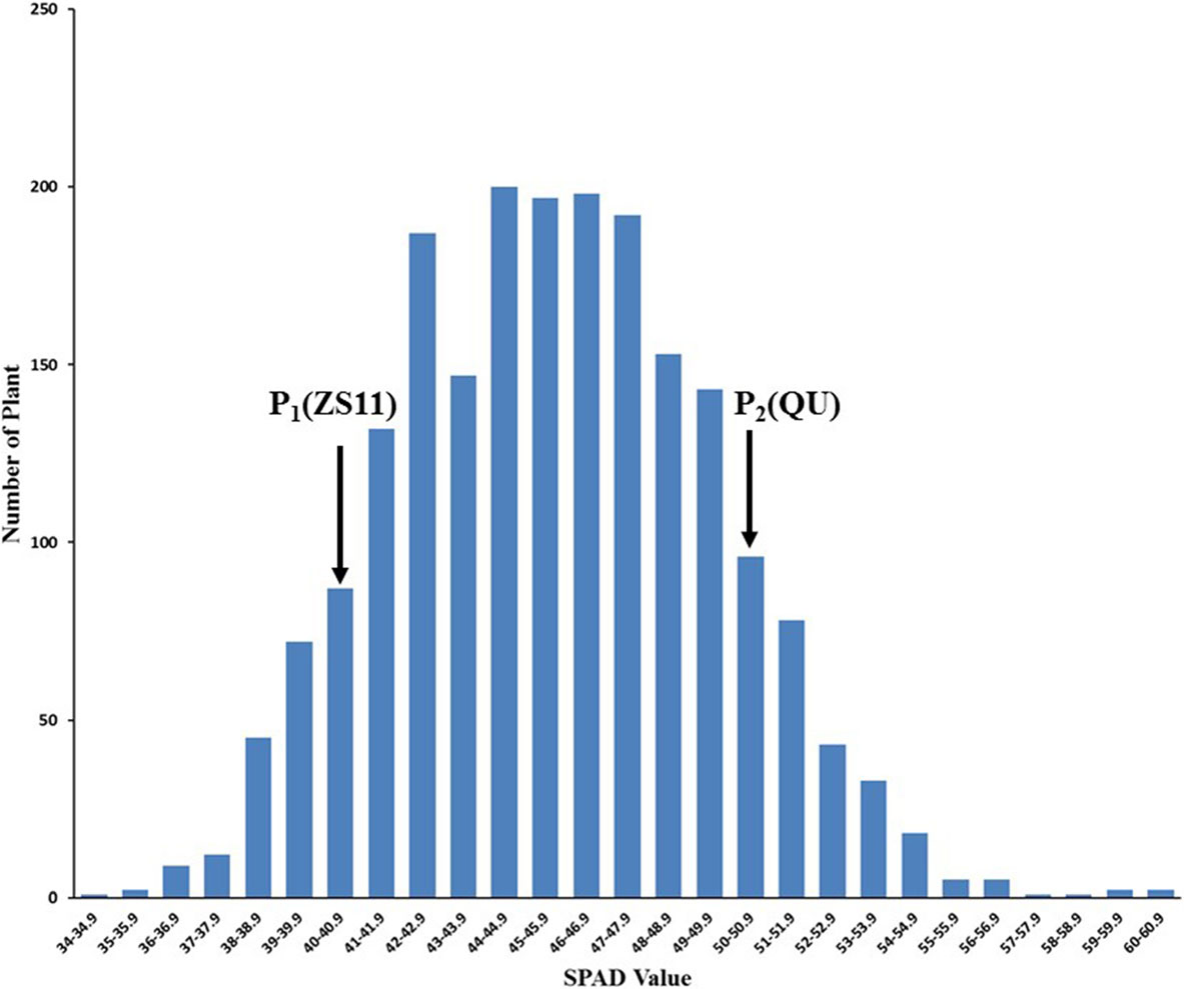
Phenotypic frequency distribution of chlorophyll SPAD in the BC_4_F_2_ population. The lowest point of the column chart represents the dividing value (SPAD = 43). Leaves were considered to have high chlorophyll content(*n* = 1514) with a SPAD value≥43 and a low chlorophyll content(*n* = 547) with a SPAD value < 43. A chi-square test showed that the segregation pattern of the chlorophyll content trait was in keeping with the expected Mendelian segregation ratio of 3:1 (χ2 = 2.53)

The first fully developed leaves counting from the tops of the 2061 individuals in the BC_4_F_2_ population at the six-leaf stage were measured by SPAD (SPAD 502, Japan). The distribution of chlorophyll content in the BC_4_F_2_ population was bimodal, with the lowest point of the column chart as the dividing line (SPAD = 43). SPAD≥43 was regarded as the high chlorophyll content (*n* = 1514), and SPAD<43 was regarded as the low chlorophyll content (*n* = 547). A chi-square test showed that the segregation pattern of the chlorophyll content trait was in keeping with the expected Mendelian segregation ratio of 3:1 (χ^2^ = 2.53) (high-chlorophyll content vs. low-chlorophyll content) (Fig. 1). Among the random selection of 198 individuals in the BC_6_F_1_ population, the markers SSR2 and Indel100 were used to validate the effect of *cqSPDA2*. The result of a chi-square test was in keeping with a 1:1 (χ^2^ = 1.46; χ^2^ = 1.82) (AA:Aa) Mendelian ratio (Additional file 1: Table S1).

### Fine mapping of *cqSPDA2*

To fine-map the *cqSPDA2* locus and identify the candidate genes, 87 primer pairs of indel markers were designed to uniformly cover the preliminary mapping interval (A02 21.87–22.91 Mb). As a result, 28 polymorphic markers were detected in the two parental lines and selected DH lines. Twenty-three of these markers were found to cosegregated in the DH lines. The 469 individuals in the BC_3_F_2_ population were then analyzed using these indel primers. The linkage map constructed using the indel data and corresponding chlorophyll content phenotypes showed that 15 of the indel primers were tightly linked to the *cqSPDA2* locus (Additional file 2: Table S2). The *cqSPDA2* locus was delimited to an interval of 5.2 cM between Indel3 and Indel15 (Additional file 3: Fig. S1). The fragments amplified by the primers Indel3, Indel6, Indel15 and Indel17 near the QTL were recovered. TA cloning was performed with the PMD18-T vector, and the physical location of the region was found to be within the 188 kb range of 21.88–22.07 Mb (Additional file 4: Fig. S2).

**Fig. 2:**
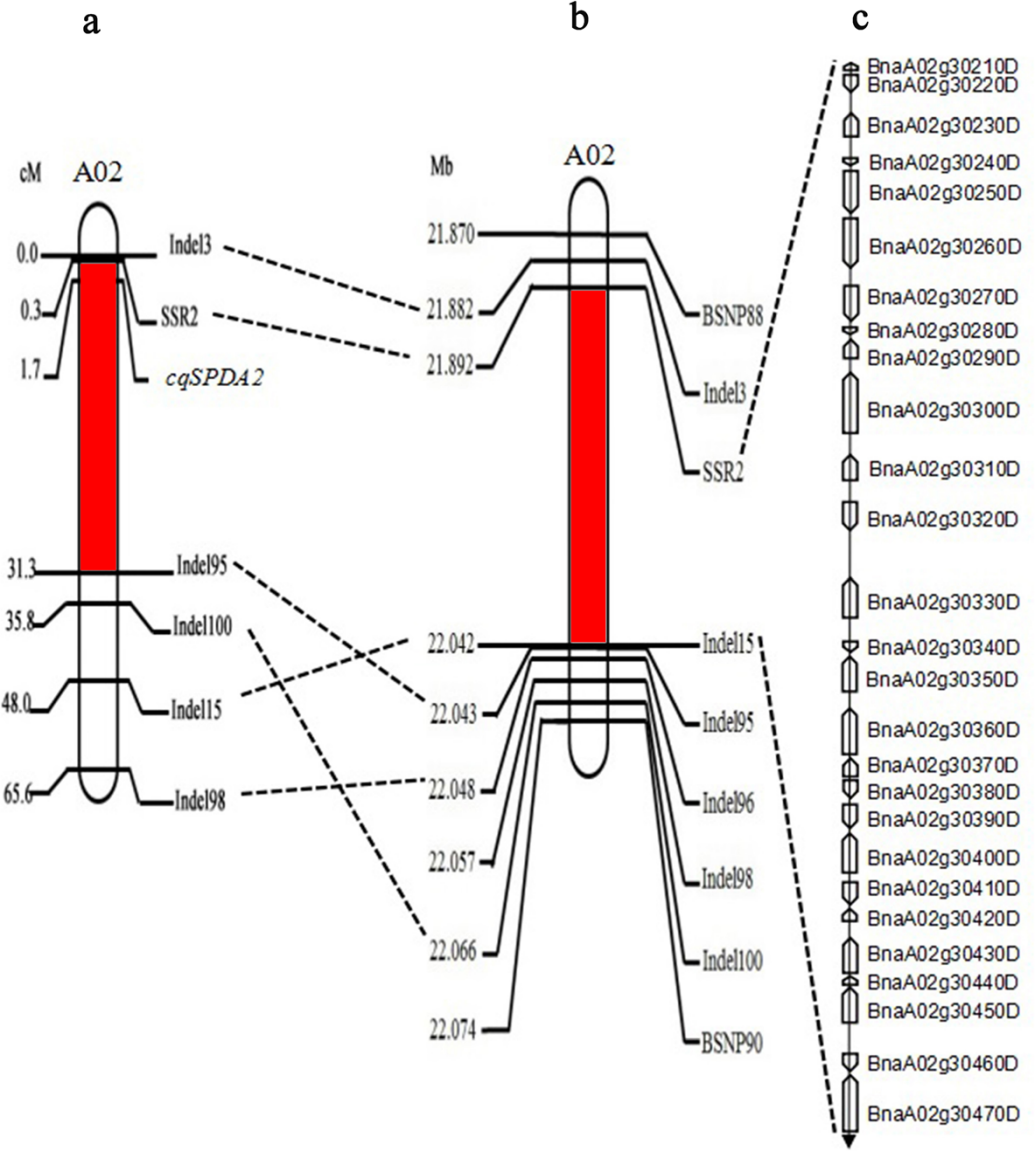
Genetic and physical maps of the *cqSPDA2* gene locus and candidate gene analysis. **a** Genetic linkage map of the *cqSPDA2* region on chromosome A02. The numbers between the markers indicate genetic distance in centimorgans. **b** Fine mapping of the *cqSPDA2* locus in the BC_4_F_2_ population. The *cqSPDA2* was narrowed down to a 152-kb interval between the markers SSR2 and Indel15. The numbers between the markers indicate physical distance. **c** Genetic and physical maps of the candidate genes in the targeted interval and the annotated genes in the *Brassica napus* genome annotation database (http://www.genoscope.cns.fr/brassicanapus/)

**Fig. 3:**
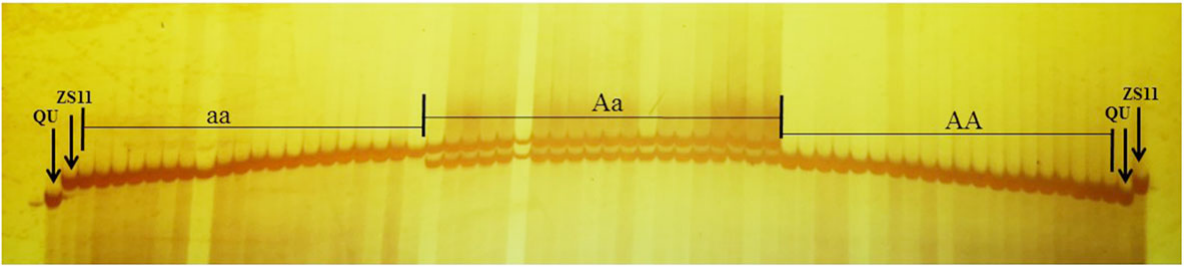
Codominant marker and closely linked to *cqSPDA2*. The aa group (low-chlorophyll phenotype): the mean of SPAD = 40.0 ± 0.27; the Aa group (medium-chlorophyll phenotype): the mean of SPAD = 44.8 ± 1.44; the AA group (high chlorophyll phenotype): the mean of SPAD = 53.3 ± 0.80

Next, 16 indel and 19 SSR primers were designed within the new, narrow mapping interval, and the fragments amplified from 5 primers were polymorphic and tightly linked to the *cqSPDA2* locus (Additional file 5: Table S3). These new primers helped to narrow the interval for the 250 individuals in the BC_4_F_2_ population. As a result, the *cqSPDA2* locus was mapped to a 152 kb interval between SSR2 and Indel15 (Fig. 2). BSNP88 and BSNP90 were developed kompetitive allele-specific PCR (KASP) markers based on SNP analysis in theis interval (Additional file 6: Table S4). SSR2 is a codominant marker closely linked to *cqSPDA2*. Twenty BC_4_F_2_ plants with low-, medium- and high-chlorophyll phenotypes were selected, and their SSR2 genotypes were determined. The results showed that the three groups of different phenotypes can be divided into three genotypes: AA (dominant homozygous, high- chlorophyll phenotype), Aa (heterozygote, medium-chlorophyll phenotype) and aa (recessive homozygous, low-chlorophyll phenotype). These results suggested that SSR2 was closely linked to *cqSPDA2* and could be effectively used in marker-assisted selection (MAS) (Fig. 3).

### Quantitative RT-PCR of genes in the mapping interval

**Fig. 4:**
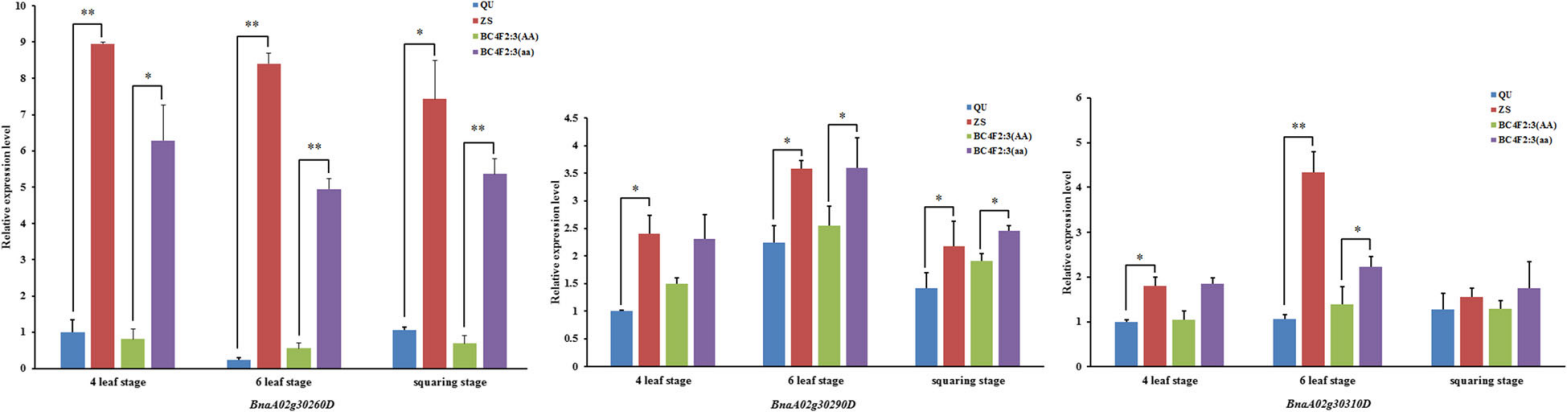
Differential expression of 3 genes in the mapping interval in the parents and NILs. The relative expression levels were calculated by the 2^−△△Ct^ method based on the QU samples, and three replicates were performed for each sample. The housekeeping gene Actin7 was used as the internal control. Values shown are means ± SD (*n* = 3). Student’s t-test was used to compare QU with ZS11 and BC_4_F_2:3_ (AA) with BC_4_F_2:3_ (aa). * denotes significance at the probability level of 0.05. ** denotes significance at the probability level of 0.01

According to the *Brassica napus* genome annotation database (http://www.genoscope.cns.fr/ brassicanapus/), twenty-seven genes were identified in the targeted mapping interval of 21.89–22.04 Mb on A02 (Additional file 7: Table S5). Quantitative real-time PCR (qRT-PCR) was used to identify the expression levels of the genes in the targeted mapping interval between the leaves of different genotypes. The melting and amplification curves of twenty-seven genes were analyzed, and the results showed that 24 primer pairs could be used to analyze gene expression (Additional file 8: Table S6). Thus, twenty-four genes in the mapping interval and the housekeeping gene *Actin7* were quantified by qRT-PCR (Additional file 9: Table S7). The results showed that the expression levels of the three genes (*BnaA02g30260D*, *BnaA02g30290D* and *BnaA02g30310D*) were all higher in ZS11 and BC_4_F_2:3_(aa) than in QU and BC_4_F_2:3_(AA) at three stages. Student’s t-test was used to compare QU with ZS11 and BC_4_F_2:3_ (AA) with BC_4_F_2:3_ (aa). *BnaA02g30290D and BnaA02g30310D* showed significant differences in expression between BC_4_F_2:3_(AA) and BC_4_F_2:3_(aa) at the 6-leaf stage (*p* < 0.05), and the difference in *BnaA02g30260D* was highly significant (*p* < 0.01) (Fig. 4). There was no consistent expression difference in the other genes tested among QU, BC_4_F_2:3_(AA), ZS11 and BC_4_F_2:3_(aa) at the three stages. Therefore, *BnaA02g30260D, BnaA02g30290D* and *BnaA02g30310D* were likely candidate genes for *cqSPDA2*.

### Agronomic traits analysis

To investigate the effect of *cqSPDA2* on agronomic traits, 50 plants with the AA genotype (high-chlorophyll content) and 50 plants with the aa genotype (low-chlorophyll content) were selected from the BC_4_F_2_ population by molecular marker and SPAD analysis. We investigated plant height, silique length, number of seeds per silique, number of siliques per plant, 1000-grain weight, and individual plant yield. The results showed that plant height, number of seeds per silique, number of siliques per plant and individual plant yield were highly significantly different between the AA-genotype plants and the aa-genotype plants (*P* < 0.01). There was a difference in 1000-grain weight but no difference in silique length between groups (*P* < 0.05) (Table 1).

**Table 1:** Agronomic trait comparisons of AA-genotype plants and aa-genotype plants in the BC_4_F_2_ population

Trait	AA genotype plant	aa genotype plant
Plant height (cm)	192.016 ± 11.70	182.534 ± 14.23^b^
Silique length (cm)	94.234 ± 10.91	92.672 ± 4.35
Seeds per silique	280.06 ± 28.98	263.88 ± 19.86^b^
1000-grain weight(g)	3.6388 ± 0.47	3.8256 ± 0.42^a^
Yield per plant(g)	21.58 ± 10.39	13.3122 ± 8.08^b^
Siliques per plant	274.84 ± 111.19	162.08 ± 83.81^b^

Fifty AA-genotype plants with high chlorophyll content and 50 aa- genotype plants with low chlorophyll content were selected by molecular markers and SPAD. Student’s t-test was used to compare the AA- and aa-genotype plants. ^a^indicates significance at the 0.05 probability level; ^b^indicates significance at the 0.01 probability level. Data are shown as mean ± SD (*n* = 50 for each sample)

## Discussion

### Significance and QTL analysis of chlorophyll content

The leaf is the main photosynthetic organ in plants, and its chlorophyll content is an important agronomic trait for crop yield. Ninety to 95 % of plant dry matter is produced by photosynthesis, and crop yield is primarily derived from the photosynthetic products of leaves [24]. Chlorophyll is an important pigment involved in photosynthesis in chloroplasts, which can absorb and transform light energy, and an important index used to evaluate leaf photosynthetic capacity [25]. Increasing crop yield by increasing chlorophyll content is one of the important objectives of high-light-efficiency breeding [26]. The emergence of high-throughput sequencing technology provides a new method for the development of molecular markers. Molecular marker technology promotes rapid genetic map construction, and provides convenient data for fine mapping. Chlorophyll content is a quantitative characteristic that is primarily controlled by nuclear genes and has high heritability. At present, research on chlorophyll QTLs has been performed in different populations of various crops, such as rice [12, 14, 16, 17], wheat [13, 15], soybean [27, 28], and cabbage [18], and especially rice [17]. Previous studies have examined the location of chlorophyll in the embryo [19] and pericarp [20] of winter *Brassica napus*., In addition, QTLs were detected under drought and salt stress conditions, and a candidate gene related to salt tolerance was predicted [21–23]. Wang et al. [11] detected a QTL on chromosome C08 in *Brassica napus* using chlorophyll deficient mutants by measuring the absolute chlorophyll content in leaves.

### Fine mapping using NILs and MAS

The development of NILs is a productive strategy for fine mapping and evaluating genetic effects during chlorophyll QTL studies [29]. In this study, a NIL population for the *cqSPDA2* regions was constructed using ZS11 as the recurrent parent with flanking markers. The flanking markers’ Indel1, 3, 15, 87 were used to carry out foreground selection for construction the population of BC_3_F_2_ and BC_4_F_2_ populations, and the mapping interval was reduced to 152 kb (Fig. 2). This strategy for mapping the target genes is reasonable, inexpensive, and highly efficient. Based on BC_4_F_2_ phenotype and BC_6_F_1_ genotype analysis, the segregation of chlorophyll content was consistent with chi-square test (Fig. 1 and Additional file 1: Table S1). These results indicated that *cqSPDA2* is the dominant QTL controlling chlorophyll content in *Brassica napus*. The NIL analysis also showed positive correlations between *cqSPDA2* and agronomic traits such as yield, plant height, seeds per silique and siliques per plant (Table 1).

Molecular marker SSR2 with close linkage was developed through fine mapping (Fig. 3). Indel15 was also next to *cqSPDA2*, these markers could accelerate the breeding process for molecular marker assisted selection in the future.

### Candidate genes prediction

According to qRT-PCR analysis, *BnaA02g30260D*, *BnaA02g30290D* and *BnaA02g30310D* were suitable candidate genes for *cqSPDA2* among the twenty-four genes annotated in the *Brassica napus* genome in the mapping interval (Additional file 9: Table S7). *BnaA02g30260D*, which is part of a disease-resistance protein family, has transmembrane receptor activity, nucleoside-triphosphatase activity, nucleotide binding funtion, and ATP binding function and is involved in signal transduction, defense response, apoptosis, and innate immune response according to its annotations. Further study is needed to determine whether *BnaA02g30260D* affects chlorophyll synthesis. *BnaA02g30290D* is FK506- and rapamycin-binding protein 15 kD-2 (*FKBP15–2*) and has peptidyl-prolyl cis-trans isomerase activity related to protein folding. Luan et al. [30] found that *AtFKBP15–1* and *AtFKBP15–2* had the highest homology to *FKBP13* and encoded functional homologs of *FKBP13*. *AtFKBP13* was reported to be associated with Rieske protein both before and after the import of proteins into the chloroplast stroma, and *AtFKBP13* can play a role in the downregulation of Rieske protein accumulation. Rieske is a subunit of the cytochrome b_6_f complex, which is one of the four complexes of the photosynthetic electron transport chain [31]. It was also reported that when *ScFKBP12* was transferred into *Arabidopsis*, chloroplast formation and the expression of genes related to chloroplast formation were inhibited [32]. In this study, the expression levels of *BnaA02g30290D* (*AtFKBP15–2*) in the NIL (aa) and ZS11 plants were all higher than those in the NIL (AA) and QU at the three stages; if this gene inhibited the formation of chlorophyll, this finding would be consistent with the abovementioned results. *BnaA02g30310D* is homologous to *GCH-1* in *Arabidopsis thaliana*. *GCH-1* is the first enzyme in tetrahydrobiopterin (BH4) biosynthesis [33]. BH4 is an essential coenzyme for all three kinds of nitric oxide synthase (NOS) [34]. *AtNOA1* (*AtNOS1*) is located in *Arabidopsis* chloroplasts, and *OsNOA1* (*OsNOS1*) is also located in rice chloroplasts [35–37]. Yang et al. [37] found that the chlorophyll content decreased with increasing *OsNOA1* at a low temperature (22 °C). He [38] suggested that *OsNOA1* directly regulates chloroplast-encoded proteins by affecting the function of the chloroplast ribosome and then transmits a signal to the nucleus through the chloroplast retrograde signaling pathway mediated by Mg protoporphyrin IX, further affecting the expression of chloroplast proteins encoded by the nuclear genes. The location of this study, Qinghai Province, is on the Qinghai-Tibet Plateau, where the average temperature during crop growth is low. In this location, the expression level of *BnaA02g30310D* (*GCH-1*) in NIL (aa) and ZS11 was high at the three stages observed, especially at the six-leaf stage, which is consistent with the results obtained by He [37, 38].

Additional experiments, such as transgenic complementation test, CRISPR/Cas9, VIGS and RNAi, are warranted to investigate whether *BnaA02g30260D*, *BnaA02g30290D* and *BnaA02g30310D* are the genes underlying *cqSPDA2*. Analysis of the regulatory network controlling chlorophyll synthesis will facilitate molecular breeding of *Brassica napus* for high yield.

## Conclusions

In this study, we constructed NILs and narrowed the interval of *cqSPDA2* to 152 kb on A02 between SSR2 and Indel15. According to the *Brassica napus* genome annotation database, there were twenty-seven genes in this mapping interval. *BnaA02g30260D*, *BnaA02g30290D* and *BnaA02g30310D* were identified as suitable candidate genes for *cqSPDA2* according to a qRT-PCR analysis, and thus, these genes may be responsible for chlorophyll synthesis. In addition, the dominant locus *cqSPDA2* has positive effects on agronomic traits.

## Methods

### Plant materials

**Fig. 5:**
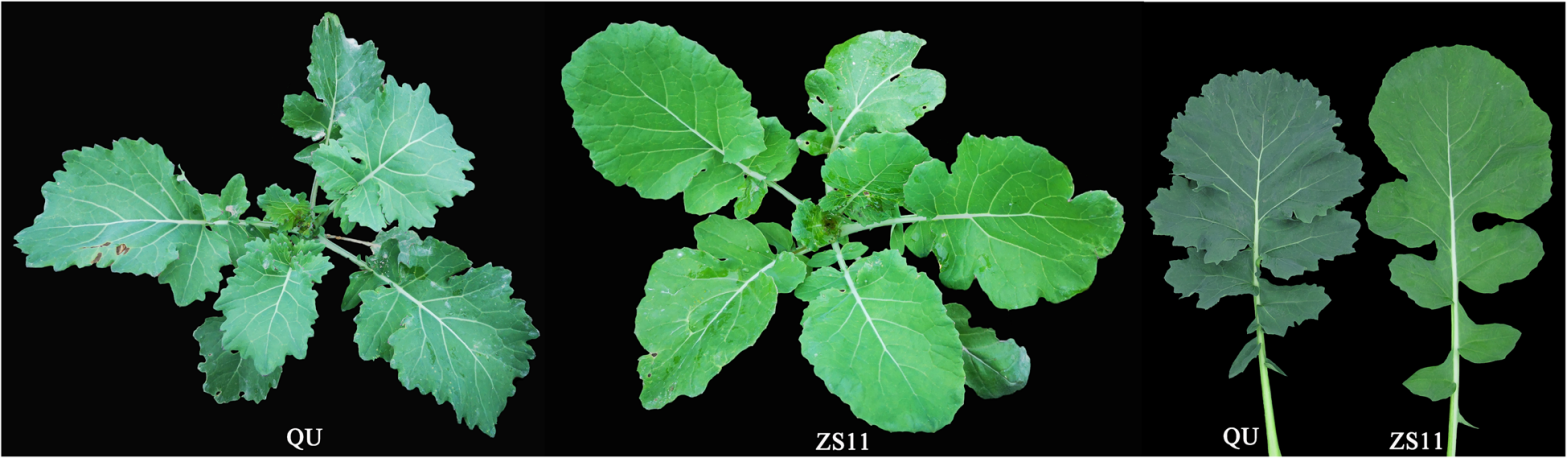
Leaf color phenotypes of the parents QU and ZS11. QU: deep green leaves, high- chlorophyll content, SPAD value = 50.4; ZS11: light green leaves, low-chlorophyll content, SPAD value = 40.6

The leaves of ZS11 have low chlorophyll content, and the leaves of QU have high chlorophyll content (Fig. 5). To obtain a relatively simple genetic background and to fine-map *cqSPDA2*, we constructed an NIL population. An F_1_ line with the QU genotype in the *cqSPDA2* region was selected and backcrossed to ZS11 for three generations. BC_3_F_1_ individuals were selfed to generate a BC_3_F_2_ mapping population backcrossed to ZS11. The flanking markers Indel1 and Indel87 were used to construct the NIL population with foreground selection (Additional file 2: Table S2).

The BC_4_F_1_ individuals with a QU genetic background in the *cqSPDA2* region as selected by the flanking markers Indel3 and Indel15 (Additional file 3: Fig. S1) were selfed to generate a BC_4_F_2_ population, which was used for fine mapping of the *cqSPDA2* locus. The detailed process of population development is illustrated in Additional file 10: Fig. S3. BC_4_F_2:3_ individuals with the AA genotype (homozygous for *cqSPDA2*) and aa genotype (without *cqSPDA2*) were detected by the flanking markers Indel3 and Indel15 and subjected to qRT-PCR analysis. In addition, each population was grown in the experimental plots with a spacing of 30 cm (between rows) × 15 cm (within rows). The BC_3_F_2_ and BC_4_F_2_ populations were grown at the same density in fields in Yuanmou, Yunnan (altitude 898 m, 101°52′N, 25°42′E) and Xining, Qinghai (altitude 2225 m, 101°49′N, 36°34′E), respectively. The BC_4_F_2:3_ and BC_6_F_1_ populations were grown in a greenhouse at the Academy of Agricultural and Forestry Sciences, Qinghai University (Xining, Qinghai, China). Standard crop management practices were followed.

### Phenotypic trait and data analysis

The testing targets included every plant of the population that was lacking diseases and insect pests. According to the previous method of chlorophyll determination, we measured three positions in the distal third of the first fully developed leaf from the top of each plant at the six-leaf stage by SPAD (SPAD 502, Japan) during the 9:00–11:00 am interval [39]. Each measurement was repeated three times per leaf, and the veins were avoided. Statistical analysis was performed with Excel. Chi-square tests were performed on the segregation data to determine the genetic regulation of the chlorophyll content.

### DNA extraction and development of molecular markers development

Total DNA was extracted from fresh leaves using the CTAB method [40]. PCR was performed in a 20-μL reaction solution containing 2 μL DNA, 2 μL 2 mM dNTPs, 2 μL 10× PCR buffer, 1 μL Taq, 1 μL of 2 μM forward and reverse primers and 12 μL of ddH_2_O. The PCR program was carried out according to Yang’s method with minor modifications [41]. The PCR products were separated on 6% nondenatured polyacrylamide gels and detected by silver staining [42]. Indel (insertion/deletion) markers were developed from the resequencing data of the parents according to the *B. napus* ‘Darmor-bzh’ reference genome sequence in the primary mapping interval. SSR markers were developed based on the *B. napus* ‘Darmor-bzh’ reference genome sequence corresponding to the interval. The sequences of the SSR markers were designed using SSR Hunter 1.3 and Primer Premier 5.0 [43, 44].

### Mapping of the *cqSPDA2* locus

The BC_3_F_2_ and BC_4_F_2_ family populations were used to fine map the *cqSPDA2* locus using indel and SSR markers. First, we designed 87 indel markers in the primer mapping interval (21.87–22.91 Mb on chromosome A02) to map the *cqSPDA2* locus. A linkage map for the *cqSPDA2* locus was constructed using JoinMap 4.0 [45]. The mapping interval for the *cqSPDA2* locus was gradually reduced using the mapping results of the BC_3_F_2_ population. Finally, additional indel and SSR markers within the new narrow mapping interval were designed to fine-map the *cqSPDA2* locus based on the BC_4_F_2_ population with WinQTLCart 2.5. The physical location was obtained by blasting the *Brassica napus* genome database using the indel and SSR sequences. The physical linkage map was produced with MapDraw 2.1 [46].

### TA cloning

The specific markers closely linked to *cqSPDA2* were sequenced by NIL population scanning. Specific fragments were collected according to Yi et al. [47]. The product was ligated into the PMD18-T vector (Takara), and the transformed clone was detected with M13 primers. Six positive clones were randomly selected and sequenced by Sangon Biotech (Shanghai) Co., Ltd. [48].

### Genes in the mapping interval

All genes within the targeted mapping interval on A02 were identified using annotations from the *Brassica napus* genomes (http://www.genoscope.cns.fr/brassicanapus/) and annotated according to the BRAD annotations (http://brassicadb.org/brad/blastPage.php). The homologous sequences were aligned using BLASTN (http://blast.ncbi.nlm.nih.gov/).

### RNA extraction and qRT-PCR analysis

BC_4_F_2:3_ (91 AA-genotype plants homozygous for *cqSPDA2* and 104 aa-genotype plants without *cqSPDA2*) were grown in a greenhouse in September 2019. Total RNA was isolated from the first fully developed leaves counted from the top of each plant (at the 4-leaf stage, 6-leaf stage and squaring stage) of the BC_4_F_2:3_ population and the parental lines using TRNzol-A+ Total RNA Reagent (Takara, Dalian, China) according to the manufacturer’s protocol. RNA integrity was monitored using 1% agarose gel electrophoresis. cDNA was obtained via reverse transcription of total RNA using the PrimeScript RT Reagent Kit (Takara, Dalian, China) and following the manufacturer’s instructions.

We performed a qRT-PCR analysis to identify the genes in the mapping interval. Real-time PCR was conducted using LightCycler 480 II 96-Well PCR Plates (Roche, Rotkreuz, Switzerland). The utilized reaction system contained 10 μL of 2 × SG Fast qPCR Master Mix (B639271, BBI), 2 μL cDNA, and 10 μM gene-specific primers in a final volume of 20 μL. The thermal cycling conditions used were 95 °C for 3 min, followed by 45 cycles at 95 °C for 5 s and 60 °C for 30 s followed by a final extension stage. The relative expression levels of all the genes in the mapping interval were calculated by the 2^−△△Ct^ method based on the QU samples and three replicates were performed for each sample [49]. The housekeeping gene Actin7 was used as the internal control to calculate the relative expression levels of each gene. Three biological replicates were performed in this experiment and t-test was used for statistical analysis.

### Phenotyping for agronomic traits

To evaluate the agronomic efficiency of *cqSPDA2*, 100 individuals (50 AA- and 50 aa- genotype plants) from the BC_4_F_2_ population were sampled using the markers Indel3 and Indel15, and their chlorophyll content was characterized. The agronomic traits investigated were as follows: plant height (cm), total siliques per plant, silique length (cm), seeds per silique, 1000-grain weight (g), and yield per plant (g). The mean values, standard deviations and significance analyses of all the agronomic traits were compared between the AA- and aa-genotype plants by Minitab16 and Excel2010.

## Supplementary Information

**Additional file 1: Table S1.** Genotyping of *cqSPDA2* in BC_6_F_1_ population.

**Additional file 2: Table S2.** Primer sequences designed in this study.

**Additional file 3: Fig. S1.** Genetic linkage map of *cqSPDA2*.

**Additional file 4: Fig. S2.** The local genetic linkage map and physical map of *cqSPDA2* on chromosome A02 in BC_3_F_2_ population.

**Additional file 5: Table S3.** Primer sequences designed in this study.

**Additional file 6: Table S4.** Primer sequences of KASP designed in this study.

**Additional file 7: Table S5.** Genes in the mapping interval on the chromosome A02 and their orthologs in *Arabidopsis*.

**Additional file 8: Table S6.** Primers designed for qRT-PCR of genes in the mapping interval.

**Additional file 9: Table S7.** Gene expression in the mapping interval on A02.

**Additional file 10: Fig. S3.** The scheme of NIL development for fine mapping.

## Data Availability

All data used during the study are included in this published article and its additional files.

## Abbreviations

QTL: Quantitative trait locus
NIL: Near-isogenic line
DH: Doubled haploid
qRT-PCR: Quantitative real-time PCR
SNP: Single nucleotide polymorphism
Indel: Insertion-deletion
SSR: Simple sequence repeat
KASP: kompetitive allele-specific PCR
SPAD: Soil and plant analyzer development
*FKBP*: FK506- and rapamycin-binding protein
*GCH-1*: GTP cyclohydrolase І
BH4: Tetrahydroboipterin
NOS/NOA: nitric oxide synthase
