# Supplementary material for: Fine mapping of the QTL cqSPDA2 for chlorophyll content in Brassica napus L
Source: BMC Plant Biol. 2020 Nov 9;20:511. doi: 10.1186/s12870-020-02710-y (PMC7654151; doi:10.1186/s12870-020-02710-y)
Supplement: Supplementary file 1 — Additional file 1: Table S1. Genotyping of cqSPDA2 in BC6F1 population. [file 12870_2020_2710_MOESM1_ESM.pdf]

Additional file 1: Table S1. Genotyping of *cqSPDA2* in BC_6F_1 population.

Marker	AA	aa	total	Expectation	χ^2	P value
SSR2	90	108	198	1:1	1.46	0.73
Indel100	89	109	198	1:1	1.82	0.91

AA indicates the number of plant from the homozygous dominant genotype with *cqSPDA2*; aa indicates the number of plant from homozygous recessive genotype without *cqSPDA2*
