# Supplementary material for: Fine mapping of the QTL cqSPDA2 for chlorophyll content in Brassica napus L
Source: BMC Plant Biol. 2020 Nov 9;20:511. doi: 10.1186/s12870-020-02710-y (PMC7654151; doi:10.1186/s12870-020-02710-y)
Supplement: Supplementary file 2 — Additional file 2: Table S2. Primer sequences designed in this study. [file 12870_2020_2710_MOESM2_ESM.pdf]

Additional file 2: Table S2. Primer sequences designed in this study.

Marker	Marker Type	Forward primer (5'-3')	Reverse primer (5'-3')
**Indel1**	**InDel**	**AAATCTACATCACACCAAAGC**	**GCCTTGTTGGGTCTTTTAT**
**Indel3**	**InDel**	**GTCCACACCAAATCCACTAC**	**TGCTAGGAGAAACAACCTGT**
**Indel6**	**InDel**	**ACATACTTTGATGCCTTGGT**	**ACTCTGAGGTTTTCAAGCAG**
**Indel15**	**InDel**	**AACATCGCCATTGTAAGTCT**	**CAACTGCAACACAACAATTT**
**Indel17**	**InDel**	**AACAAAAGATCATTGCTTTCA**	**CATGGATATCACCCTGAGAA**
**Indel19**	**InDel**	**GCTGTTGTTGTCTGTGACAT**	**AAACACCTCTCCTCCTCTTC**
**Indel48**	**InDel**	**AAAGTAACAGACACATGGACG**	**CTGAAAGGTGAGAAATGGAG**
**Indel52**	**InDel**	**TTCCCAGAGTCAGAAGAAGA**	**CTCCTTATCTATGGAGCACG**
**Indel56**	**InDel**	**TGCTTTCAGTAGCAAGGATT**	**ACAAACCAATCATTCGAGTC**
**Indel59**	**InDel**	**TGTTTGTTTGAAAAAGCTCA**	**AAACCAAGAAAAACAAGGAA**
**Indel63**	**InDel**	**GTATACACCAATTTCGGGTC**	**CATCCAATGCTTCTCTTTCT**
**Indel75**	**InDel**	**CAGCAATAATCCAAGTCACA**	**GGTTAAACGATACTAACCGC**
**Indel77**	**InDel**	**CAGTGGTAGACCTCCACAAT**	**GCAAGACATATGATCCTTTCA**
**Indel86**	**InDel**	**AATGATACCAACCCACCAC**	**TGGAAGGTATAAAGTCTCATCC**
**Indel87**	**InDel**	**TCTTTCTGCTCAAAGGTTGT**	**AGGGTTTAAAAGTCCCAAC**
Indel2	InDel	TATAAAAGACCCAACAAGGC	GTAGTGGATTTGGTGTGGAC
Indel4	InDel	TAATAAGGCCATAAGCATCC	CTCCCTTGAGAAATGAAAAA
Indel10	InDel	TAACTCAAGTCTCCCCGTTA	GAGAAACAAGACCGTGAAGA
Indel24	InDel	TTGGGAGAGCGAAATAAATA	AGTATGCGCTTTTGATTTTT
Indel37	InDel	CGCCATAGATTCTCAGTTCT	GATGTGAGGGAAAATAAACG
Indel38	InDel	TTTTTATTGGGTCCCTTTTT	TTCAAAACCATTCTTCTTTCA
Indel41	InDel	CAATCCTCCTTATCACCTCA	CAGATATAAGCGGAGCAATC
Indel44	InDel	AGGAACCAAATGAGAAGTCA	AAAAATCATCAACCAATCAAA
Indel61	InDel	TATGCGTATGCTGTTTCATC	CATAACTGCAACATGCCTAA
Indel70	InDel	ACCGAAAAATTATTTAGTATGA	AGAATAGGTGATTAATCGTGA
Indel73	InDel	ATTACGCGATCATTGTTTTT	TAGAATGCCTTTCTTTCGAG
Indel76	InDel	AAGATCGGAGAATGAAAACA	TCTAGAACGATCGGCTACAT
Indel81	InDel	AAAGTTCGACAAAATTGGTG	CAATGATTCGTACTTCCATGT

15 markers that tightly linked with the *cqSPDA2* locus are displayed in bold.
