# Supplementary material for: Fine mapping of the QTL cqSPDA2 for chlorophyll content in Brassica napus L
Source: BMC Plant Biol. 2020 Nov 9;20:511. doi: 10.1186/s12870-020-02710-y (PMC7654151; doi:10.1186/s12870-020-02710-y)
Supplement: Supplementary file 3 — Additional file 3: Fig. S1. Genetic linkage map of cqSPDA2. [file 12870_2020_2710_MOESM3_ESM.pdf]

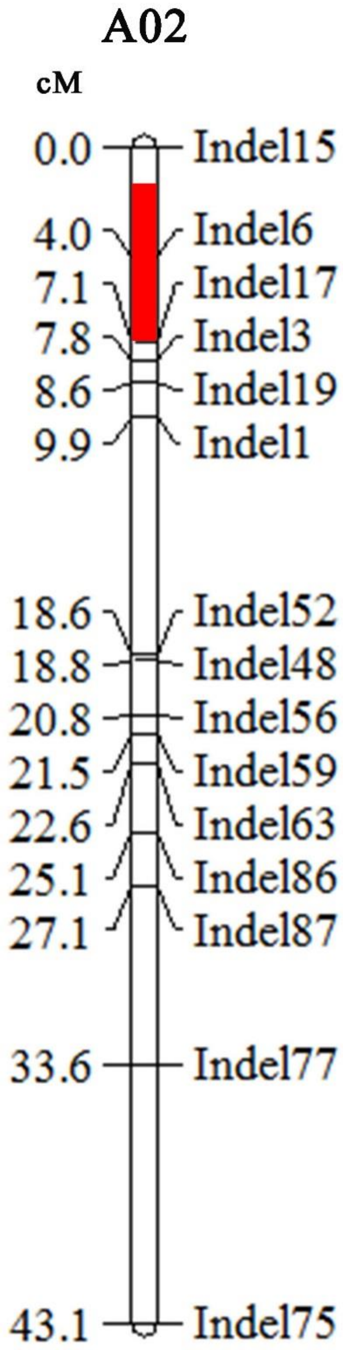

Additional file 3: Figure S1 Genetic linkage map of *cqSPDA2*. The numbers on the left of the chromosome A02 represent the marker’s geneticdistance (cM). Red box indicates QTL confidence interval.
