# Supplementary material for: Fine mapping of the QTL cqSPDA2 for chlorophyll content in Brassica napus L
Source: BMC Plant Biol. 2020 Nov 9;20:511. doi: 10.1186/s12870-020-02710-y (PMC7654151; doi:10.1186/s12870-020-02710-y)
Supplement: Supplementary file 4 — Additional file 4: Fig. S2. The local genetic linkage map and physical map of cqSPDA2 on chromosome A02 in BC3F2 population. [file 12870_2020_2710_MOESM4_ESM.pdf]

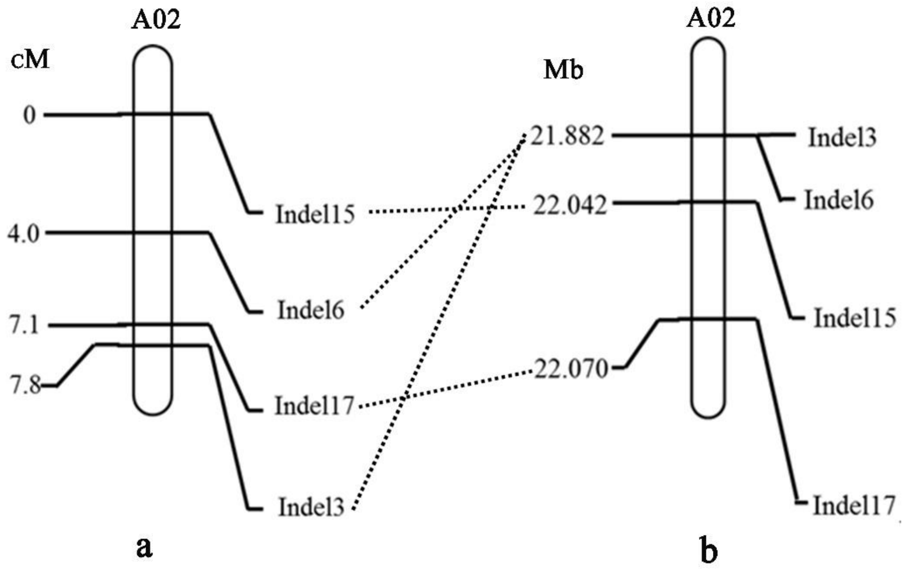

Additional file 4: Figure S2 The local genetic linkage map and physical map of *cqSPDA2* on chromosome A02 in BC_3F_2 population. **a** The local genetic linkage map. **b** The physical map.
