# Supplementary material for: Fine mapping of the QTL cqSPDA2 for chlorophyll content in Brassica napus L
Source: BMC Plant Biol. 2020 Nov 9;20:511. doi: 10.1186/s12870-020-02710-y (PMC7654151; doi:10.1186/s12870-020-02710-y)
Supplement: Supplementary file 5 — Additional file 5: Table S3. Primer sequences designed in this study. [file 12870_2020_2710_MOESM5_ESM.pdf]

Additional file 5: Table S3. Primer sequences designed in this study.

Marker	Marker Type	Forward primer (5'-3')	Reverse primer (5'-3')
SSR2	SSR	CATGCCACAAAGATCAATCG	ATCTGGTTCACGGGTGTCTC
Indel95	InDel	TCATCTCATGCAACAATGTC	CCTCTTGGTGCTACAGGTTA
Indel96	InDel	GAAAAGATATCGACTGTGCC	AATGCAGATTAGCTTCTCCA
Indel98	InDel	GGAAGTCTCAGCTCAGTTTG	CCACACATCCACAATCTACA
Indel100	InDel	GAGTCAAGAAAGACCATTGC	TTGATTTCTTTCTCTTGGTTG
Indel15	InDel	AACATCGCCATTGTAAGTCT	CAACTGCAACACAACAATTT
Indel3	InDel	GTCCACACCAAATCCACTAC	TGCTAGGAGAAACAACCTGT
