# Supplementary material for: Fine mapping of the QTL cqSPDA2 for chlorophyll content in Brassica napus L
Source: BMC Plant Biol. 2020 Nov 9;20:511. doi: 10.1186/s12870-020-02710-y (PMC7654151; doi:10.1186/s12870-020-02710-y)
Supplement: Supplementary file 6 — Additional file 6: Table S4. Primer sequences of KASP designed in this study. [file 12870_2020_2710_MOESM6_ESM.pdf]

Additional file 6: Table S4. Primer sequences of KASP designed in this study.

Marker	Sequence
BSNP88F1	GAAGGTCGGAGTCAACGGATTAGAACTCCCTTGAGAAATGAAAA
BSNP88F2	GAAGGTGACCAAGTTCATGCTAGAACTCCCTTGAGAAATGAAAG
BSNP88R1	GGTTGTTTCTCCTAGCATCTATGA
BSNP90F1	GAAGGTCGGAGTCAACGGATTTGCACAAAGATCTGGTCTTTAATA
BSNP90F2	GAAGGTGACCAAGTTCATGCTGCACAAAGATCTGGTCTTTAATG
BSNP90R1	ATGAAGATTCTGATGCCATGG
