# Supplementary material for: Fine mapping of the QTL cqSPDA2 for chlorophyll content in Brassica napus L
Source: BMC Plant Biol. 2020 Nov 9;20:511. doi: 10.1186/s12870-020-02710-y (PMC7654151; doi:10.1186/s12870-020-02710-y)
Supplement: Supplementary file 7 — Additional file 7: Table S5. Genes in the mapping interval on the chromosome A02 and their orthologs in Arabidopsis. [file 12870_2020_2710_MOESM7_ESM.pdf]

Additional file 7: Table S5. Genes in the mapping interval on the chromosome A02 and their orthologs in *Arabidopsis*.

Gene ID	Position (bp)	Homologue in *A. thaliana*	Gene function
BnaA02g30210D	chrA02:21892059-21892443	AT5G48490.1	Bifunctional inhibitor/lipid-transfer protein/seed storage 2S albumin superfamily protein
BnaA02g30220D	chrA02:21897708-21898935	AT5G48500.1	Uncharacterized protein
BnaA02g30230D	chrA02:21905151-21907039	AT5G38220.3	Alpha/beta-Hydrolases superfamily protein
BnaA02g30240D	chrA02:21922533-21922881	AT5G48560.1	Transcription factor *bHLH78*
BnaA02g30250D	chrA02:21923207-21926710	AT4G16890.1	Disease resistance protein
BnaA02g30260D	chrA02:21926798-21930428	AT5G51630.2	Disease resistance protein
BnaA02g30270D	chrA02:21934212-21936672	AT5G48560.1	Putative *bHLH* transcription factor
BnaA02g30280D	chrA02:21936812-21936924		PREDICTED: Brassica napus transcription factor *bHLH78*-like
BnaA02g30290D	chrA02:21938935-21940337	AT5G48580.1	Peptidyl-prolyl cis-trans isomerase *FKBP15-2*
BnaA02g30300D	chrA02:21944673-21951722	AT5G51630.1	Disease resistance protein
BnaA02g30310D	chrA02:21955728-21958153	AT3G07270.2	GTP cyclohydrolase I
BnaA02g30320D	chrA02:21961765-21963075	AT5G13930.1	Chalcone synthase
BnaA02g30330D	chrA02:21979040-21981843	AT1G14800.1	Predicted protein
BnaA02g30340D	chrA02:21984480-21984810	AT5G13930.1	Chalcone synthase
BnaA02g30350D	chrA02:21985298-21987016	AT5G48630.1	Cyclin-C1-2
BnaA02g30360D	chrA02:21990969-21993198	AT5G48640.1	Cyclin family protein
BnaA02g30370D	chrA02:21993664-21994959	AT5G48655.3	RING/U-box domain-containing protein
BnaA02g30380D	chrA02:21995437-21996668	AT5G48660.1	PREDICTED: B-cell receptor-associated protein 31-like isoform X1
BnaA02g30390D	chrA02:21997365-21999287	AT5G48720.2	Protein XRI1
BnaA02g30400D	chrA02:22000709-22002975	AT5G48760.2	60S ribosomal protein L13A
BnaA02g30410D	chrA02:22004795-22006340	AT4G38830.1	Cysteine-rich receptor-like protein kinase 26

Additional file 7: Table S5. Genes in the mapping interval on the chromosome A02 and their orthologs in *Arabidopsis*. *(Continued)*

Gene ID	Position (bp)	Homologue in *A. thaliana*	Gene function
BnaA02g30420D	chrA02:22006708-22007330	AT5G44620.1	Cytochrome P450
BnaA02g30430D	chrA02:22016679-22018973	AT5G48830.1	Unknown protein
BnaA02g30440D	chrA02:22020346-22020772	AT5G48840.1	Pantoate-beta-alanine ligase
BnaA02g30450D	chrA02:22021041-22023175	AT5G48850.1	Male sterility MS5 family protein
BnaA02g30460D	chrA02:22041388-22042414	AT5G48870.1	Sm-like protein
BnaA02g30470D	chrA02:22042463-22045797	AT5G48880.3	KAT5/PKT1/PKT2 peroxisomal 3-keto-acyl-CoA thiolase
