# Supplementary material for: Fine mapping of the QTL cqSPDA2 for chlorophyll content in Brassica napus L
Source: BMC Plant Biol. 2020 Nov 9;20:511. doi: 10.1186/s12870-020-02710-y (PMC7654151; doi:10.1186/s12870-020-02710-y)
Supplement: Supplementary file 8 — Additional file 8: Table S6. Primers designed for qRT-PCR of genes in the mapping interval. [file 12870_2020_2710_MOESM8_ESM.pdf]

Additional file 8: Table S6. Primers designed for qRT-PCR of genes in the mapping interval.

Name of Primers	Sequence of primers
Actin7-F	GCTGACCGTATGAGCAAAG
Actin7-R	AAGATGGATGGACCCGAC
BnaA02g30210D-F	GAATCCAACGAACCCATCAA
BnaA02g30210D-R	AATACCGAAAGAACCGAGCA
BnaA02g30220D-F	GTTTGGAGGTTGGGACTGGA
BnaA02g30220D-R	TGAGGGTAAGGAGGGTAGTGGT
BnaA02g30230D-F	CCACCTTCTTACGCTTTCATTAC
BnaA02g30230D-R	GCTCAAACATCTGACCCAAATC
BnaA02g30240D-F	GATGATGAACGGAACGATGCT
BnaA02g30240D-R	TTGACAGGGAGACTGGAGTTAG
BnaA02g30250D-F	TAGCCGATTCCACCACCAT
BnaA02g30250D-R	TTAGCACTTCAAGATCATCCACATC
BnaA02g30260D-F	TGCGTAATCTGAAACTTCTACACTT
BnaA02g30260D-R	GTTCCTTCCCACAACTTCTCAA
BnaA02g30270D-F	AAGTCAACGGAGCAACAACAA
BnaA02g30270D-R	CGATGAGTTCTCTGATAACGAAGC
BnaA02g30290D-F	AGTGAGAAGCGGCGATGTT
BnaA02g30290D-R	TTGGAGTGAAATGAGAGAGAAGAC
BnaA02g30300D-F	TCCCATCGTATGATCTGGCTAT
BnaA02g30300D-R	TTCTCCACCCACTCCTCCTT
BnaA02g30310D-F	CCATCTGGTGTCGCTGTTGT
BnaA02g30310D-R	GCTCCGAACTTTCATCCTCAA
BnaA02g30330D-F	TTCACATCTACACGCCTATCCTT
BnaA02g30330D-R	TACCACCACCACTGCTTGT
BnaA02g30340D-F	ATGAGGAGGAAGGCAGTAGAG
BnaA02g30340D-R	AGTCCGTCCAGTTGACATCT
BnaA02g30350D-F	TGTAACCTATATGCGCCGTGT
BnaA02g30350D-R	AGATACAAGCAAGTGGGAGCA
BnaA02g30360D-F	TGGACCTCATTCTTGTACACCC
BnaA02g30360D-R	ACCATGCAGTGATGTCTTTCTCT
BnaA02g30370D-F	TGAGAGCAACACGTGGCAT
BnaA02g30370D-R	GGAACAGGCTGAGAAGAAGGTA
BnaA02g30380D-F	TGTTCGTGGAGGGTGCTATT
BnaA02g30380D-R	CATCTTCATCTGGTCAAGGCTT
BnaA02g30390D-F	CCTGTAGCGAGTCGGGTTAT
BnaA02g30390D-R	AAAGGGTTGTTTCCATAGGCT
BnaA02g30400D-F	TCAACGGGCAGAAGGTAGTC
BnaA02g30400D-R	AGGAGCACGAAAGTGAATAGGT
BnaA02g30410D-F	GACTGGTAGGAGAATGAAGAACTT
BnaA02g30410D-R	GCAGGTAGGAGAACACATAACT
BnaA02g30420D-F	TCGGTGGTACGGATACATCA
BnaA02g30420D-R	GCGGTGAGGATTAAGGAGTG
BnaA02g30430D-F	TGATTCTGACACTGCGATGACT
BnaA02g30430D-R	CAAGCTCAGGTCATAGCAGGA
BnaA02g30450D-F	TCAGATGGTTGAGCCAGACG
BnaA02g30450D-R	TAGCCGACGAGGTAACGAAG

Additional file 8: Table S6. Primers designed for qRT-PCR of genes in the mapping interval. *(Continued)*

Name of Primers	Sequence of primers
BnaA02g30460D-F	GGTGCATAGGGTCGAAGATAAG
BnaA02g30460D-R	TAAACGTCGAAGCCTTTGAGA
BnaA02g30470D-F	ACCTTGCAGGACTCAAAGTTAGC
BnaA02g30470D-R	CCAATAGCAATGGCTCCTCC
