# Supplementary material for: Fine mapping of the QTL cqSPDA2 for chlorophyll content in Brassica napus L
Source: BMC Plant Biol. 2020 Nov 9;20:511. doi: 10.1186/s12870-020-02710-y (PMC7654151; doi:10.1186/s12870-020-02710-y)
Supplement: Supplementary file 9 — Additional file 9: Table S7. Gene expression in the mapping interval on A02. [file 12870_2020_2710_MOESM9_ESM.pdf]

Additional file 9: Table S7. Gene expression in the mapping interval on A02.

		BnaA02g30210D	BnaA02g30220D	BnaA02g30230D	BnaA02g30240D	BnaA02g30250D	BnaA02g30260D	BnaA02g30270D	BnaA02g30290D
4-leaf stage	QU[a]	1	1	1	1	1	1	1	1
	ZS11[b]	17.6494568	1.3505903	1.36363693	2.75499998	0.76040653	8.95383502	1.74038905	2.40528789
	$BC_4F_{2:3}$(AA)[c]	3.53396463	1.18319532	0.66638786	2.47095822	0.40086701	0.8052987	1.63652724	1.4923762
	$BC_4F_{2:3}$(aa)[d]	0.48933639	1.27355549	1.17724768	5.02551873	0.59835695	6.28334716	1.7064745	2.31934263
6-leaf stage	QU[a]	16.2390714	2.17385387	4.10540044	1.74067205	1.35380267	0.23493046	1.74901782	2.24732631
	ZS11[b]	2.93254936	1.81244546	1.9577692	3.84007519	0.77664362	8.40234753	1.72938158	3.58795434
	$BC_4F_{2:3}$(AA)[c]	18.6670292	2.46192481	2.19552402	2.61473857	0.99717215	0.56200934	2.09554922	2.55406526
	$BC_4F_{2:3}$(aa)[d]	21.1069919	2.07989673	1.85392328	3.07668286	1.14621484	4.94143563	2.06129199	3.59382363
budding stage	QU[a]	4.01521263	1.34845042	0.48968383	35.1033088	2.23613725	1.05538218	3.00163819	1.41623127
	ZS11[b]	40.4448793	2.06173897	0.76041871	3.70317788	0.32858491	7.43624565	0.99899488	2.17428479
	$BC_4F_{2:3}$(AA)[c]	22.4629718	1.7762724	1.17313065	1.72086264	0.70586734	0.68854592	2.24290234	1.91339903
	$BC_4F_{2:3}$(aa)[d]	25.3817143	1.67699937	1.49320088	1.67871908	0.61388725	5.36661967	2.14367124	2.46236186

Additional file 9: Table S7. Gene expression in the mapping interval on A02. *(Continued)*

		BnaA02g30300D	BnaA02g30310D	BnaA02g30330D	BnaA02g30340D	BnaA02g30350D	BnaA02g30360D	BnaA02g30370D	BnaA02g30380D
4-leaf stage	QU[a]	1	1	1	1	1	1	1	1
	ZS11[b]	5.87162084	1.80022872	0.45151048	3.07247479	2.56165019	1.34120235	1.16869237	1.83326353
	BC4F2:3(AA)[c]	0.9574239	1.04374082	0.1468235	2.19006861	1.78408093	1.20899937	1.4273856	1.77954522
	BC4F2:3(aa)[d]	5.38977366	1.84747355	0.36712583	2.08761457	3.73451683	0.87018395	1.81011696	1.469689
6-leaf stage	QU[a]	0.45755105	1.0666536	0.88857199	2.96063273	2.6962526	0.76181276	1.32446887	1.75469628
	ZS11[b]	7.20027221	4.3385437	0.46757108	3.28618429	2.81433317	1.32912691	2.31797359	1.93931699
	BC4F2:3(AA)[c]	0.89917614	1.3888694	0.16543537	4.79618047	2.96191512	1.03966016	2.02371357	2.47059421
	BC4F2:3(aa)[d]	3.36953093	2.23411359	0.19118352	5.94963834	3.60042352	1.23700882	2.28549088	2.53779552
budding stage	QU[a]	2.40622805	1.26744045	222.291143	10.3311031	2.11886391	2.31016968	3.28741449	2.65606111
	ZS11[b]	1.46214542	1.55049512	11.0433294	2.69451053	1.49956607	2.02461459	1.16295125	2.12223443
	BC4F2:3(AA)[c]	0.62155637	1.29499745	0.27049774	4.25734515	2.36363238	1.31166693	1.80883773	2.12508571
	BC4F2:3(aa)[d]	2.76159414	1.75925846	0.37492042	4.46238167	2.8534598	1.38038813	1.74600484	2.4606394

Additional file 9: Table S7. Gene expression in the mapping interval on A02. *(Continued)*

		BnaA02g30300D	BnaA02g30310D	BnaA02g30330D	BnaA02g30340D	BnaA02g30350D	BnaA02g30360D	BnaA02g30370D	BnaA02g30380D
4-leaf stage	QU[a]	1	1	1	1	1	1	1	1
	ZS11[b]	5.87162084	1.80022872	0.45151048	3.07247479	2.56165019	1.34120235	1.16869237	1.83326353
	BC4F2:3(AA)[c]	0.9574239	1.04374082	0.1468235	2.19006861	1.78408093	1.20899937	1.4273856	1.77954522
	BC4F2:3(aa)[d]	5.38977366	1.84747355	0.36712583	2.08761457	3.73451683	0.87018395	1.81011696	1.469689
6-leaf stage	QU[a]	0.45755105	1.0666536	0.88857199	2.96063273	2.6962526	0.76181276	1.32446887	1.75469628
	ZS11[b]	7.20027221	4.3385437	0.46757108	3.28618429	2.81433317	1.32912691	2.31797359	1.93931699
	BC4F2:3(AA)[c]	0.89917614	1.3888694	0.16543537	4.79618047	2.96191512	1.03966016	2.02371357	2.47059421
	BC4F2:3(aa)[d]	3.36953093	2.23411359	0.19118352	5.94963834	3.60042352	1.23700882	2.28549088	2.53779552
budding stage	QU[a]	2.40622805	1.26744045	222.291143	10.3311031	2.11886391	2.31016968	3.28741449	2.65606111
	ZS11[b]	1.46214542	1.55049512	11.0433294	2.69451053	1.49956607	2.02461459	1.16295125	2.12223443
	BC4F2:3(AA)[c]	0.62155637	1.29499745	0.27049774	4.25734515	2.36363238	1.31166693	1.80883773	2.12508571
	BC4F2:3(aa)[d]	2.76159414	1.75925846	0.37492042	4.46238167	2.8534598	1.38038813	1.74600484	2.4606394

[a] Male parent with *cqSPDA2*. [b] Female parent without *cqSPDA2*. [c] The homozygous dominant genotype with *cqSPDA2*. [d] The homozygous recessive genotype without *cqSPDA2*. The relative expression levels were calculated by the $2^{-\triangle\triangle Ct}$ method based on the samples of QU and three replicates for each sample. The housekeeping gene Actin7 was used as the internal control.
