# Supplementary figures and images for: Fine mapping of the QTL cqSPDA2 for chlorophyll content in Brassica napus L
Source: BMC Plant Biol. 2020 Nov 9;20:511. doi: 10.1186/s12870-020-02710-y (PMC7654151; doi:10.1186/s12870-020-02710-y)

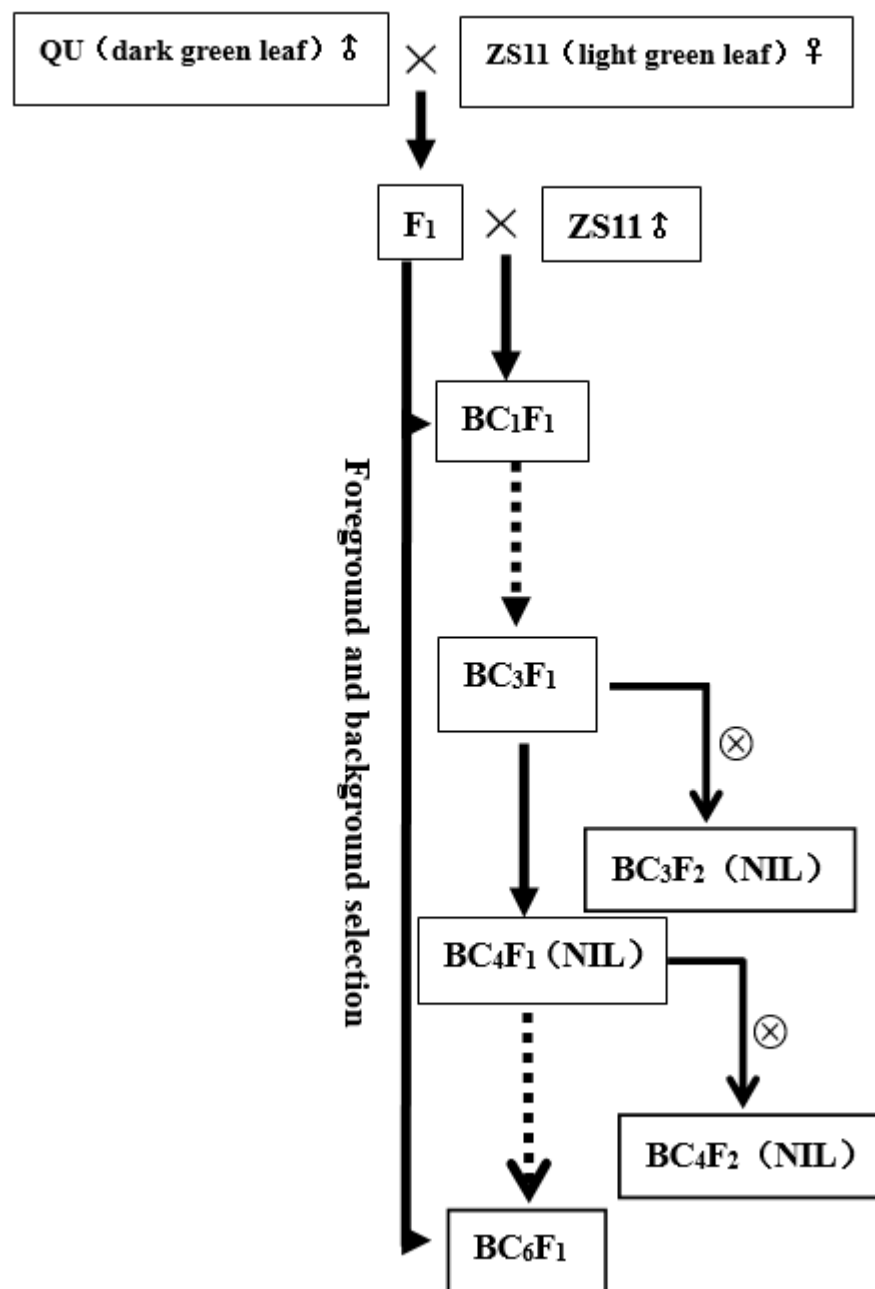

**Additional file 10: Figure S3** The scheme of NIL development for fine mapping.

Supplement: Supplementary file 10 — Additional file 10: Fig. S3. The scheme of NIL development for fine mapping. [file 12870_2020_2710_MOESM10_ESM.pdf]
